# Performance of Radiological and Biochemical Biomarkers in Predicting Radio-Symptomatic Knee Osteoarthritis Progression

**DOI:** 10.3390/biomedicines12030666

**Published:** 2024-03-16

**Authors:** Ahmad Almhdie-Imjabbar, Hechmi Toumi, Eric Lespessailles

**Affiliations:** 1 Translational Medicine Research Platform, PRIMMO, University Hospital Center of Orleans, 45100 Orleans, France; hechmi.toumi@chu-orleans.fr (H.T.); eric.lespessailles@chu-orleans.fr (E.L.); 2 Department of Rheumatology, University Hospital Center of Orleans, 45100 Orleans, France

**Keywords:** trabecular bone texture, knee osteoarthritis, conventional radiograph, X-rays, prediction of knee osteoarthritis progression, longitudinal variations, biochemical biomarkers

## Abstract

Imaging biomarkers permit improved approaches to identify the most at-risk patients encountering knee osteoarthritis (KOA) progression. This study aimed to investigate the utility of trabecular bone texture (TBT) extracted from plain radiographs, associated with a set of clinical, biochemical, and radiographic data, as a predictor of long-term radiographic KOA progression. We used data from the Foundation for the National Institutes of Health (FNIH) Biomarkers Consortium dataset. The reference model made use of baseline TBT parameters adjusted for clinical covariates and radiological scores. Several models based on a combination of baseline and 24-month TBT variations (TBT∆TBT) were developed using logistic regression and compared to those based on baseline-only TBT parameters. All models were adjusted for baseline clinical covariates, radiological scores, and biochemical descriptors. The best overall performances for the prediction of radio-symptomatic, radiographic, and symptomatic progression were achieved using TBT∆TBT parameters solely, with area under the ROC curve values of 0.658 (95% CI: 0.612–0.705), 0.752 (95% CI: 0.700–0.804), and 0.698 (95% CI: 0.641–0.756), respectively. Adding biochemical markers did not significantly improve the performance of the TBT∆TBT-based model. Additionally, when TBT values were taken from the entire subchondral bone rather than just the medial, lateral, or central compartments, better results were obtained.

## 1. Introduction

Bone is among the key factors in the pathological process of OA, as illustrated by the numerous features depicted by several imaging modalities, such as Magnetic Resonance Imaging (MRI)-based bone marrow lesions [1], dual-energy X-ray absorptiometry (DXA)-based tibial subchondral bone mineral density [2], X-ray-based subchondral bone thickening, osteophytes [3], and the trabecular bone texture (TBT) of subchondral tibial bone [4].

TBT is a promising imaging biomarker for the prediction of radiographic knee osteoarthritis (KOA) outcomes (incidence, progression, and total knee arthroplasty) [5,6,7,8,9,10]. TBT-based prediction models have, furthermore, demonstrated to be robust and flexible, as they have been trained and validated on different cohorts [11].

Searching for tools for predicting KOA progression is not limited to imaging biomarkers, as KOA is also characterized by an imbalance between pro-inflammatory (procatabolic) and anti-inflammatory cytokines and growth factors. Thus, the corresponding imbalance between tissue degradation and formation can be assessed and monitored by both synovial and blood-based biomarkers. Biochemical trial enrichment biomarkers have been suggested to improve successful therapy development for KOA [8,12].

In the present study, we included biochemical data obtained from the Foundation for the National Institutes of Health (FNIH) OA Biomarkers Consortium, Bethesda, MD, USA, namely urinary C-terminal crosslinked telopeptide type II collagen (uCTX-II), crosslinked N-telo peptide of type I collagen (sNTXI) serum, and hyaluronic acid (sHA) serum, which have previously been evaluated for their role in the progression of the disease [8,13,14]. The restriction to utilizing these three biochemical biomarkers stemmed from their demonstrated superior predictive capacity within the FNIH dataset [8]. Combining imaging biomarkers and molecular biomarkers has received limited attention [15] despite its potential to improve the prediction of KOA progression, and to stratify therapeutic interventions [16,17]. Thus, in addition to the baseline parameters employed by the reference model (age, sex, Body Mass Index (BMI), Kellgren–Lawrence (KL), joint space narrowing in the medial tibial plateau (JSNM), and TBT at baseline), we examined the use of longitudinal TBT variations associated with the aforementioned biochemical markers (uCTX-II, sNTXI, and sHA) for the prediction of the radiographic, symptomatic, and radio-symptomatic progression of KOA in the FNIH dataset.

## 2. Materials and Methods

### 2.1. Patients

This study included data from 600 patients (one knee per patient) obtained from the nested case–control study of the Osteoarthritis Initiative (OAI) cohort, previously identified by the FNIH Biomarkers Consortium [7]. The OAI permission group of the National Institute of Mental Health Data Archive, Bethesda, MD, USA, gave us access to the raw data used in our study. Only one knee per patient was included to avoid inter-organ correlation [18]. We recall that the inclusion criteria imposed by the FNIH dataset included clinical, biological, and radiological descriptors of included patients whose knees had KL scores of 1–3 at baseline and whose relevant information concerning medial knee OA progression was available at 24-month and 48-month control points.

### 2.2. Definition of Radiographic and Symptomatic Progression

Radiographic progression was defined as a loss in medical joint space width of less than 0.7 mm during the first 24 months of the study, but a loss of at least 0.7 mm during the period from 24 months to 48 months, while symptomatic progression was defined by an insignificant worsening of pain during the first 24 months but a significant worsening of pain during the period from 24 months to 48 months of the study, expressed by an increase in the Western Ontario McMaster Universities Osteoarthritis (WOMAC) pain score of at least 9 points (on a 0–100 normalized score) [19]. Radio-symptomatic progressors were defined as patients with both radiographic and symptomatic progression. Non-progressors were defined as patients with no radiographic progression in the medial or lateral tibial plateaus, and no symptomatic progression in both the index and contralateral knees.

One participant with missing BMI data at baseline and two participants with missing biochemical data were excluded from our study. Of the remaining 597 knees, 192 were both radiographic and symptomatic (radio-symptomatic) progressors (Group 1), 102 were radiographic-only progressors (Group 2), 103 were symptomatic-only progressors (Group 3), and 200 were non-progressors (Group 4). 

### 2.3. Trabecular Bone Texture Analysis

**Figure 1:**
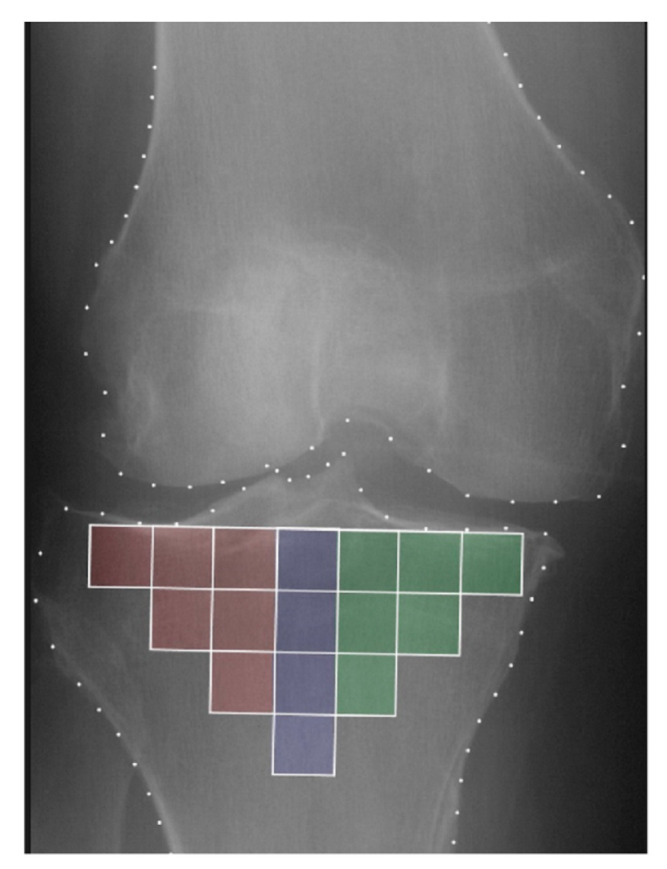
Illustration of the 16 regions of interest (ROIs) automatically selected, covering the entire tibial subchondral bone structure. Medial, central, and lateral ROIs are highlighted in green, blue, and red, respectively.

Several well-known approaches have been used to describe the fractal dimension of XR image texture, including fractal signature analysis [20], the Whittle estimator [9], and the quadratic variation method (VAR) [9,10]. These three various fractal analysis techniques all produced reliable outcomes in terms of their ability to predict KOA progression [10]. The VAR approach, used in [6,11], was retained for the studies presented in this paper. As previously reported [11], the cut-off scale was observed around 500 mm on the empirical variograms. From each knee X-ray, the TBT parameters were calculated from a patchwork of 16 ROIs covering the entire tibial subchondral bone structure (Figure 1). Two fractal parameters were extracted, corresponding to the texture complexity computed for the two micro (μ: below 400 mm) and milli (m: above 600 mm) scales of observation, and filtered in both horizontal (HF) and vertical (VF) directions, yielding 64 descriptors for each patchwork.

### 2.4. Biochemical Parameters

We included in our prediction models the biochemical data (BIO: uCTX-II, sNTXI, and sHA) obtained from the FNIH OA Biomarkers Consortium.

### 2.5. Prediction Models

As described earlier, the reference model involved the use of baseline clinical covariates (CLIN: age, sex, and BMI), radiological readings (KL and JSNM), and TBT descriptors, whereas the proposed model involved, in addition, the use of longitudinal variations in TBT (∆TBT) parameters. All models were developed using nested logistic regression [5,7,10,21] to evaluate their prediction performance based on the TBT parameters of the complete tibial subchondral bone structure (Models 1–5), or those of the medial tibial plateau (TBTM) (Model 6), lateral tibial plateau (TBTL) (Model 7), or central tibial plateau (TBTC) (Model 8). Figure 1 shows the location of these regions. All prediction models were evaluated using a 10-fold cross-validation, repeated 300 times, to avoid overfitting.

- Model 1: TBT ← CLIN + KL + JSNM (Reference model)
- Model 2: ∆TBT ← CLIN + KL + JSNM
- Model 3: TBT + ∆TBT ← CLIN + KL + JSNM
- Model 4: TBT + ∆TBT
- Model 5: TBT + ∆TBT ← BIO + CLIN + KL + JSNM
- Model 6: TBTM + ∆TBTM ← CLIN + KL + JSNM
- Model 7: TBTL + ∆TBTL ← CLIN + KL + JSNM
- Model 8: TBTC + ∆TBTC ← CLIN + KL + JSNM

The model with the descriptor on the left of (←) is adjusted for the descriptor(s) on the right of (←).

### 2.6. Statistical Analysis

AUC was used as the preferred metric to determine the most predictive model [22,23]. Other statistical metrics (positive predictive value (PPV), negative predictive value (NPV), and balanced accuracy (BACC)) were also used to further evaluate the performance of the different models studied [6,24]. 

The Akaike Information Criterion (AIC) method was employed to select the most appropriate set of TBT parameters, ensuring the maintenance of good predictive performance in the proposed prediction models while reducing the risk of overfitting. Furthermore, the DeLong method was used to assess whether the difference in AUC between the proposed models and the reference model was statistically significant. Specifically, the test statistic derived from DeLong’s method facilitated the computation of the *p*-value [25]. In this study, a given model is deemed statistically significant compared to the reference model if the *p*-value is less than 0.05.

Inspired by [7], the primary analysis evaluated the ability of the 64 TBT parameters and their variations over 24 months to predict KOA radio-symptomatic progression (knees with both radiographic and symptomatic progression (Group 1; 193 progressors) compared to knees without both radiographic and symptomatic progression (Groups 2, 3 and 4; 406 controls). The secondary analyses included 4 different scenarios:

- Scenario 1 evaluated the proposed models to predict any progression (knees with either radiographic or symptomatic progression, or both (Groups 1, 2, and 3; 397 progressors) compared to knees without any progression (Group 4; 200 controls).
- Scenario 2 evaluated the proposed models to predict all progression (knees with either radiographic or symptomatic progression (Groups 2 and 3; 205 progressors) compared to knees without any progression (Group 4; 200 controls).
- Scenario 3 evaluated the proposed models to predict radiographic progression (knees with radiographic-only progression (Group 2; 102 progressors) compared to knees without radiographic progression (Groups 3 and 4; 303 controls).
- Scenario 4 evaluated the proposed models to predict symptomatic progression (knees with symptomatic-only progression (Group 3; 102 progressors) compared to knees without radiographic progression (Groups 2 and 4; 303 controls).

## 3. Results

Table 1 represents the characteristics of the knees included in the current study for the three FNIH sub-datasets: radio-symptomatic, radiographic-only, and symptomatic-only progressors, as well as the non-progressor sub-dataset.

### 3.1. Primary Analysis: Radio-Symptomatic Progression

**Figure 2:**
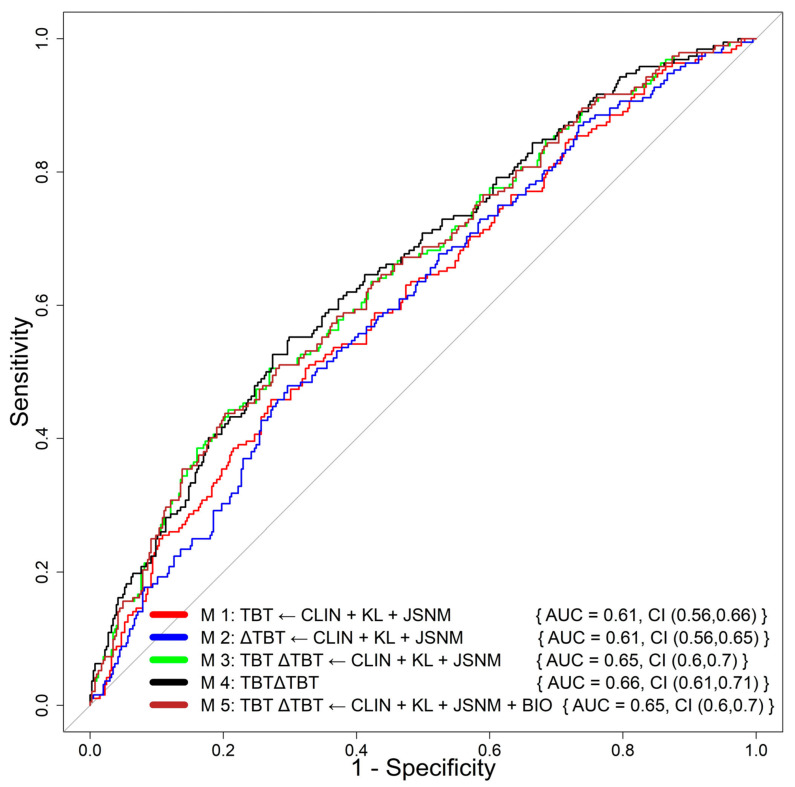
ROC curves obtained by the different models for the prediction of radio-symptomatic progression using TBT parameters of the complete tibial subchondral bone structure, adjusted for clinical, biochemical, and radiological parameters.

We first evaluated the performance of the different models for the prediction of radio-symptomatic progression using data from Group 1 (192 progressors) and Groups 2, 3, and 4 (405 controls). In this scenario, using solely TBT and ∆TBT (Model 4) statistically significantly improved the performance of the prediction of radio-symptomatic progression with an AUC = 0.658, compared to the reference model using baseline-only TBT (Model 1), which achieved an AUC of 0.613 (*p*-value = 0.03; see Figure 2). In addition, as shown in Table 2, adding BIO parameters to Model 4 very slightly improved the balanced accuracy (BACC = 0.59), positive predictive value (PPV = 0.52), and negative predictive value (NPV = 0.73) values. However, they both achieved the same AUC. 

Regarding the influence of the regions of interest (ROIs) selected for the calculation of TBT, the use of the complete subchondral zone (Model 3) provided higher performance (AUC = 0.650) compared to using medial-only (Model 6: AUC = 0.594), lateral-only (Model 7: AUC = 0.577), or central-only (Model 8: AUC = 0.572) ROIs (Table 2). More details are reported in Table S1 of the Supplementary File.

### 3.2. Secondary Analysis

#### 3.2.1. Any Progression

In this scenario, the performance of the different models was evaluated for the prediction of radiographic, symptomatic, or both progressions using data from Groups 1, 2, and 3 (397 progressors) and Group 4 (200 controls). The AUC score of Model 3, based on baseline TBT and its 24-month variation, was significantly higher (AUC = 0.679; *p*-value = 0.009) than the one obtained by Model 1, based solely on baseline TBT (AUC = 0.628). Model 3 furthermore achieved the best BACC (0.60) and PPV (0.72) values, while Model 5 with biochemical parameters, in addition, achieved slightly higher NPV (0.53) values (Table 3). In this scenario, we noticed that using the whole subchondral zone (Model 3) provided better performance than using medial-only (AUC = 0.614), lateral-only (AUC = 0.596), or central-only ROI (AUC = 0.580) compartments. Further information can be found in Table S2 of the Supplementary File.

#### 3.2.2. All Progression

In this scenario, the performance of the aforementioned models was evaluated for the prediction of radiographic or symptomatic progression using data from Groups 2 and 3 (205 progressors) and Group 4 (200 controls). Model 4, adjusted in addition to biochemical parameters, obtained the best AUC score (AUC = 0.691, *p*-value = 0.018), significantly higher than the one obtained by the reference model (AUC = 0.628). The best values were obtained by Model 4, adjusted in addition to biochemical parameters and JSNM scores, for the other statistical metrics (BACC = 0.64, PPV = 0.65, and NPV = 0.64). More details can be found in Table S3 of the Supplementary File.

#### 3.2.3. Radiographic Progression

In this scenario, the performance of the abovementioned models was evaluated for the prediction of radiographic-only progression using data from Group 2 (102 progressors) in addition to Groups 3 and 4 (303 controls). In this scenario, all knees with radiographic and symptomatic progression were excluded. The best overall performance was achieved by Model 4, adjusted in addition to clinical and biochemical parameters (Model 4B), with an AUC of 0.783, BACC of 0.66, PPV of 0.52, and NPV of 0.82, while the reference model achieved a significantly lower AUC of only 0.709 (*p*-value = 0.009), and lower values for BACC, PPV, and NPV (0.55, 0.47, 0.77, respectively). In line with the other scenarios, higher performance was achieved using the TBT parameters extracted from the complete subchondral bone, compared to using the TBT parameters extracted from the medial, lateral, or central compartments. Further information can be found in Table S4 of the Supplementary File.

#### 3.2.4. Symptomatic Progression

In this scenario, all knees with radiographic and symptomatic progression were also excluded. The performance of the different models was evaluated for the prediction of symptomatic-only progression using data from Group 3 (103 progressors) and Groups 2 and 4 (302 controls). As found in the previous scenario, the best overall performance was achieved by Model 4B with an AUC of 0.718, BACC of 0.61, PPV of 0.51, and NPV of 0.79. As for the previously mentioned scenarios, the models using the TBT parameters extracted from the complete subchondral bone achieved a higher overall performance than the models using the TBT parameters extracted from the medial, lateral, or central compartments. Further information can be found in Table S5 of the Supplementary File.

## 4. Discussion

One of the main contributions of this study is the evaluation of predictive performance gained from utilizing longitudinal variations in TBT parameters adjusted by a set of clinical, biochemical, and radiological biomarkers for predicting KOA progression. The results in the present study show that integrating both baseline and longitudinal changes in radiographic TBT descriptors plays an important role in predicting radio-symptomatic progression (best AUC = 0.658), any (radiographic, symptomatic, or both) progression (best AUC = 0.679), all (radiographic or symptomatic) progression (best AUC = 0.691), radiographic progression (best AUC = 0.718), and symptomatic progression (best AUC = 0.783).

KOA progression is often considered to be slow; 12% to 23% of knees with radiographic KOA experience radiographic progression over 5 years [26]. Hence, the findings in the current study would help in better selecting participants in future structure-modifying KOA trials. 

The use of baseline, 12-month, and 24-month TBT parameters was previously evaluated [7] for the prediction of 48-month radiographic and symptomatic progression in the FNIH cohort. In that study, the medial subchondral tibial region only was investigated to extract TBT parameters computed using the fractal signature analysis (FSA) method [20]. Introducing the time-integrated values (TIVs) of the TBT parameters over 24 months provided a benefit to the prediction of KOA radio-symptomatic progression (primary analysis), with an AUC of 0.649, compared to the use of clinical covariates alone (AUC = 0.608) [7]. While the authors in [7,27] investigated the use of the 24-month TIVs of TBT parameters, equivalent to the area under the curve defined by the baseline and 24-month TBT values, they did not investigate the use of time-longitudinal changes, quantified as the difference between the baseline and 24-month TBT values. In addition, in their study [7,27], six TBT parameters were employed, extracted from the medial tibial plateau only. 

This study also highlights the importance of exploiting the whole subchondral bone of the tibia, rather than only the medial plateau or a limited part of the medial and lateral plateaus [5,7], to extract radiographic TBT parameters as the performance of the prediction models was lower in all the different scenarios using TBT parameters extracted from the medial, lateral or central tibial plateaus alone (Tables S1–S5 of the Supplementary File).

The best AUC score was obtained for the prediction of radiographic progression (AUC = 0.783). It is more difficult to predict symptomatic (pain) progression since it is related to changes in pain scores, which are subjective due to differences in patient tolerance. In addition, it has been demonstrated that pain scores can only be considered modest markers in the prediction of KOA-related outcomes [6,11].

The results obtained by the present study confirm the interest in using both baseline TBT parameters and their variations over 24 months, allowing a better prediction of radio-symptomatic, radiographic, or symptomatic progression. When TBT descriptors were excluded, all assessed models had failed (0.5 ≤ AUC < 0.6) to poor (0.6 ≤ AUC < 0.7) values; however, when TBT descriptors were included, and their variations over 24 months were taken into account, the evaluated models’ performances improved and they were able to obtain acceptable (0.7 < AUC < 0.8) values [28,29].

Validated on the FNIH dataset, this research demonstrated the benefits of using both baseline and longitudinal changes in TBT, calculated from standardized plain knee radiographs, to improve the prediction of KOA progression within 48 months in patients with mild KOA (knees with 1 ≤ KL ≤ 3) at baseline.

In the present study, adding molecular biomarkers into the model to the core set of radiographic and clinical markers did not improve the performance of the reference model. The AUC scores of the reference model (AUC = 0.709 and 0.779 using TBT and TBT + ∆TBT, respectively) were similar to those obtained by including molecular biomarkers (AUC = 0.708 and 0.779 using TBT and TBT + ∆TBT, respectively) for the prediction of radiological progression. This observation could be because the information given by biochemical markers is already captured by data from subchondral bone (its texture), osteophytes, and joint space width, which are known as strong predictors of KOA progression [4,10,11,28,30]. Similar results have been found for the other scenarios (Table 1). The three molecular biomarkers used in the present study were selected based on their formerly successful use in the literature concerning the prediction of KOA progression [8,13]. Other relevant parameters might be used to improve KOA prediction models such as type II collagen KOA formation [12] or inter-alpha trypsin inhibitor heavy chain 1 [31]. 

Our study has several strengths. The proposed prediction models are based on TBT descriptors extracted from plain radiographs, widely used in clinical routine, while biochemical parameters are not yet included in daily clinical routines and need much more time to be extracted from blood or urine samples. Furthermore, data were selected in accordance with each specific type of progression evaluated in the current study. For radiographic-only progression, knees with symptomatic progression were not included, and vice versa; for symptomatic-only progression, knees with radiographic progression were not included. In addition, for radio-symptomatic progression, knees with symptomatic-only or radiographic-only progression were not included. To avoid possible correlation between the TBT parameters of both the baseline and 24-month variations, which can lead to problems with traditional logistic regression with respect to overfitting and convergence, the LASSO method was used as an alternative regularization method [32].

A growing number of researchers are interested in evaluating the potential of imaging biomarkers to enhance patient screening in phase III studies for KOA and determining under which conditions they provide such enhancements [4,8]. From a clinical standpoint, our model showed great precision in predicting false progressors. Incorporating such progressors in a disease-modifying osteoarthritis drug (DMOAD) randomized clinical trial could have counterproductive consequences.

## 5. Study Limitations

The limitations of this study include the absence of a femoral region of interest in our TBT analysis. The femoral subchondral bone might also provide additional information [33]. In the current study, the included imaging-based biomarkers were limited to radiography. Another limitation is the lack of investigation of the association between radiographic KOA progression and 3D MRI bone texture [34] or shape [35], or other MRI-based features such as bone marrow lesions [36]. Integrating such parameters into our model is worth assessing. Conducting a comparative analysis of the results of prediction methods based on logistic regression and those derived from alternative machine learning techniques may provide valuable insights [37]. Lastly, combining descriptors extracted from both MRI and XR images might help to improve the prediction of KOA progression [27,34]. 

Furthermore, there are several potentially interesting molecular markers for predicting KOA progression [14]. Theoretically, there is no limit to the number of biochemical markers that could be included in the model, although estimating their ability to enhance our prediction model becomes increasingly challenging.

## 6. Conclusions

In conclusion, the combination of both baseline and 24-month radiographic TBT variations can increase modestly, but significantly, the ability to predict 48-month radiographic, symptomatic, and radio-symptomatic KOA progressions. Adding the three aforementioned studied molecular markers did not significantly improve the performance of the proposed TBT∆TBT model.

## Figures and Tables

**Table 1:** Characteristics of the knees included in the current study.

	N° of Knees	Females (%)	Left Knee (%)	KL ^1^ = 1 (%)	KL = 2 (%)	KL = 3 (%)
Complete dataset	597	59.0	46.2	12.6	51.1	36.3
Radio-symptomatic progressors	192	56.8	48.4	12.5	43.2	44.3
Radiographic progressors	102	45.1	47.1	13.7	46.1	40.2
Symptomatic progressors	103	65.0	42.7	12.6	59.2	28.2
Non-progressors	200	65.0	45.5	12.0	57.0	31.0

^1^ Kellgren–Lawrence.

**Table 2:** Primary analysis: radio-symptomatic progression on trabecular bone texture parameters adjusted for clinical, biochemical, and radiographic parameters.

N°	Model	BACC	PPV	NPV	AUC (95%CI)	*p*-Value
Model 1	TBT ← CLIN + KL + JSNM *	0.55	0.50	0.70	0.613 (0.565–0.662)	-
Model 2	∆TBT ← CLIN + KL + JSNM	0.53	0.45	0.70	0.606 (0.558–0.653)	0.777
Model 3	TBT + ∆TBT ← CLIN + KL + JSNM	0.59	0.52	0.73	0.650 (0.603–0.697)	0.044
Model 4	TBT + ∆TBT	0.58	0.51	0.72	0.658 (0.612–0.705)	0.030
Model 5	TBT + ∆TBT ← BIO + CLIN + KL + JSNM	0.59	0.52	0.73	0.649 (0.601–0.696)	0.054
Model 6	TBTM + ∆TBTM ← CLIN + KL + JSNM	0.53	0.52	0.69	0.594 (0.545–0.643)	0.456
Model 7	TBTL + ∆TBTL ← CLIN + KL + JSNM	0.51	0.46	0.68	0.577 (0.527–0.627)	0.173
Model 8	TBTC + ∆TBTC ← CLIN + KL + JSNM	0.51	0.46	0.68	0.572 (0.524–0.620)	0.131

* refers to the reference model. BACC, PPV, and NPV refer to balanced accuracy, positive predictive value, and negative predictive value. TBT refers to the baseline trabecular bone texture parameters while ∆TBT refers to the variations in TBT over 24 months. CLIN and BIO refer to the baseline clinical (age, sex, and BMI) and biochemical (urine CTXII, Serum NTXI, and Serum HA) parameters, respectively. KL refers to the baseline Kellgren–Lawrence scores. JSNM refers to the baseline joint space narrowing scores in the medial tibial plateau. The model with the descriptor on the left of (←) is adjusted for the descriptor(s) on the right of (←). TBTM, TBTC, and TBTL refer to the TBT parameters extracted from the medial, central, and lateral plateaus, respectively.

**Table 3:** Secondary analysis based on TBT and ∆TBT adjusted for a set of symptomatic and radiographic parameters.

Progression	Model	BACC	PPV	NPV	AUC (95%CI)	*p*-Value
Any (Scenario 1)	TBT ← CLIN + KL + JSNM *	0.55	0.69	0.46	0.628 (0.582–0.675)	-
	TBT + ∆TBT ← CLIN + KL + JSNM	0.60	0.72	0.52	0.679 (0.634–0.724)	0.009
	TBT + ∆TBT ← BIO + CLIN + KL + JSNM	0.60	0.72	0.52	0.678 (0.633–0.723)	0.011
All (Scenario 2)	TBT ← CLIN + KL + JSNM *	0.59	0.59	0.58	0.628 (0.574–0.682)	-
	TBT + ∆TBT ← CLIN + KL + JSNM	0.63	0.64	0.63	0.684 (0.632–0.736)	0.022
	TBT + ∆TBT ← BIO + CLIN + KL + JSNM	0.63	0.64	0.62	0.684 (0.632–0.735)	0.023
Radiographic (Scenario 3)	TBT ← CLIN + KL + JSNM *	0.57	0.45	0.78	0.709 (0.653–0.765)	-
	TBT + ∆TBT ← CLIN + KL + JSNM	0.65	0.51	0.82	0.779 (0.731–0.827)	0.012
	TBT + ∆TBT ← BIO + CLIN + KL + JSNM	0.65	0.51	0.82	0.779 (0.731–0.828)	0.011
Symptomatic (Scenario 4)	TBT ← CLIN + KL + JSNM *	0.53	0.40	0.76	0.643 (0.583–0.703)	-
	TBT + ∆TBT ← CLIN + KL + JSNM	0.60	0.49	0.79	0.710 (0.654–0.766)	0.027
	TBT + ∆TBT ← BIO + CLIN + KL + JSNM	0.60	0.49	0.79	0.710 (0.654–0.766)	0.027

* refers to the reference model. BACC, PPV, and NPV refer to balanced accuracy, positive predictive value, and negative predictive value. The model with the descriptor on the left of (←) is adjusted for the descriptor(s) on the right of (←).

## Data Availability

Publicly available datasets were analyzed in this study. This data can be found online at https://nda.nih.gov/oai (accessed on 20 September 2023).

## Supplementary Materials

The following supporting information can be downloaded at: https://www.mdpi.com/article/10.3390/biomedicines12030666/s1, Table S1: Primary analysis: Prediction of radio-symptomatic progression; Table S2: Secondary analysis: Prediction of any (knees with either radiographic or symptomatic progression, or both) progression; Table S3: Secondary analysis: Prediction of all (knees with either radiographic or symptomatic progression) progression; Table S4: Secondary analysis: Prediction of radiographic progression; Table S5: Secondary analysis: Prediction of symptomatic progression.
