# Supplementary material for: Performance of Radiological and Biochemical Biomarkers in Predicting Radio-Symptomatic Knee Osteoarthritis Progression
Source: Biomedicines. 2024 Mar 16;12(3):666. doi: 10.3390/biomedicines12030666 (PMC10968173; doi:10.3390/biomedicines12030666)
Supplement: Supplementary file 1 [file biomedicines-12-00666-s001.zip › biomedicines-2895052-supplementary.pdf]

Abbreviations: BACC: Balanced accuracy; BIO: Biochemical (urinary C-terminal cross-linked telopeptides of type II collagen, crosslinked N-telopeptide of type I collagen serum, and Hyaluronic Acid serum) parameters; CLIN: clinical (age, sex, body mass index) covariates; JSNM: Joint space narrowing score in the medial tibial plateau; KL: Kellgren-Lawrence score; NPV: Negative predictive value; PPV: Positive predictive value; TBT: Trabecular bone texture; TBTC: Trabecular bone texture parameters extracted from the central compartment; TBTL: Trabecular bone texture parameters extracted from the lateral compartment; TBTM: Trabecular bone texture parameters extracted from the medial compartment.

Table S1: Primary analysis: Prediction of radio-symptomatic progression using TBT descriptors adjusted for a set of clinical, radiographic and/or biochemical parameters.

Model	BACC	PPV	NPV	AUC (95%CI)	p-value
TBT← CLIN+KL+JSNM*	0.55	0.50	0.70	0.613 (0.565 - 0.662)	-
ΔTBT← CLIN+KL+JSNM	0.53	0.45	0.70	0.606 (0.558 - 0.653)	0.777
TBT+ΔTBT← CLIN+KL+JSNM	**0.59**	0.52	**0.73**	0.650 (0.603 - 0.697)	0.044
TBT	0.55	0.53	0.70	0.609 (0.561 - 0.657)	0.697
TBT← BIO	0.55	0.50	0.70	0.617 (0.569 - 0.665)	0.630
TBT← BIO+CLIN	0.55	0.49	0.70	0.609 (0.561 - 0.658)	0.574
TBT← BIO+KL	0.55	0.51	0.70	0.615 (0.566 - 0.663)	0.859
TBT← BIO+JSNM	0.56	0.52	0.71	0.622 (0.574 - 0.671)	0.022
TBT← BIO+CLIN+KL	0.55	0.49	0.70	0.607 (0.558 - 0.656)	0.856
TBT← BIO+CLIN+JSNM	0.55	0.50	0.71	0.614 (0.566 - 0.663)	0.320
TBT← BIO+CLIN+KL+JSNM	0.55	0.49	0.70	0.613 (0.565 - 0.661)	0.287
ΔTBT	0.52	0.40	0.69	0.610 (0.562 - 0.658)	0.921
TBT+ΔTBT	0.58	0.51	0.72	**0.658** (0.612 - 0.705)	0.030
TBT+ΔTBT← BIO	**0.59**	0.52	**0.73**	**0.658** (0.611 - 0.704)	0.022
TBT+ΔTBT← CLIN	0.58	0.51	0.72	0.656 (0.609 - 0.702)	0.038
TBT+ΔTBT← KL	0.58	0.51	0.72	**0.658** (0.611 - 0.704)	0.029
TBT+ΔTBT← JSNM	0.58	0.51	0.72	**0.658** (0.612 - 0.705)	0.024
TBT+ΔTBT← BIO+CLIN	**0.59**	0.52	0.73	0.653 (0.607 - 0.700)	0.036
TBT+ΔTBT← BIO+KL	**0.59**	0.52	0.72	0.656 (0.609 - 0.702)	0.026
TBT+ΔTBT← BIO+JSNM	**0.59**	0.52	**0.73**	0.656 (0.609 - 0.702)	0.021
TBT+ΔTBT← BIO+CLIN+ KL	**0.59**	0.52	**0.73**	0.653 (0.606 - 0.699)	0.036
TBT+ΔTBT← BIO+CLIN+JSNM	**0.59**	0.52	**0.73**	0.652 (0.605 - 0.699)	0.039
TBT+ΔTBT← BIO+CLIN+KL+JSNM	**0.59**	0.52	**0.73**	0.649 (0.601 - 0.696)	0.054
TBTM← CLIN+KL+JSNM	0.51	0.42	0.68	0.571 (0.522 - 0.620)	0.062
ΔTBTM← CLIN+KL+JSNM	0.51	**0.54**	0.68	0.563 (0.513 - 0.612)	0.075
TBTM+ΔTBTM← CLIN+KL+JSNM	0.53	0.52	0.69	0.594 (0.545 - 0.643)	0.456
TBTL← CLIN+KL+JSNM	0.50	0.22	0.68	0.562 (0.514 - 0.610)	0.029
ΔTBTL← CLIN+KL+JSNM	0.50	0.38	0.68	0.555 (0.505 - 0.605)	0.026
TBTL+ΔTBTL← CLIN+KL+JSNM	0.51	0.46	0.68	0.577 (0.527 - 0.627)	0.173
TBTC← CLIN+KL+JSNM	0.50	NaN	0.68	0.511 (0.461 - 0.560)	0.000
ΔTBTC← CLIN+KL+JSNM	0.51	0.47	0.68	0.572 (0.525 - 0.620)	0.134
TBTC+ΔTBTC← CLIN+KL+JSNM	0.51	0.46	0.68	0.572 (0.524 - 0.620)	0.131

* refers to the reference model. The model with the descriptor on the left of (←) is adjusted for the descriptor(s) on the right of (←). The highest BACC, PPV, NPV and AUC values obtained are in bold.

Table S2: Secondary analysis: Prediction of any progression using TBT descriptors adjusted for a set of clinical, radiographic and/or biochemical parameters.

Model	BACC	PPV	NPV	AUC (95%CI)	p-value
TBT← CLIN+KL+JSNM*	0.55	0.69	0.46	0.628 (0.582 - 0.675)	-
ΔTBT← CLIN+KL+JSNM	0.56	0.70	0.51	0.638 (0.592 - 0.685)	0.697
TBT+ΔTBT← CLIN+KL+JSNM	**0.60**	**0.72**	0.52	**0.679** (0.634 - 0.724)	0.009
TBT	0.53	0.68	0.44	0.615 (0.568 - 0.662)	0.377
TBT← BIO	0.54	0.69	0.48	0.624 (0.577 - 0.671)	0.734
TBT← BIO+CLIN	0.54	0.69	0.48	0.617 (0.569 - 0.665)	0.303
TBT← BIO+KL	0.54	0.69	0.48	0.620 (0.573 - 0.667)	0.505
TBT← BIO+JSNM	0.54	0.68	0.46	0.626 (0.580 - 0.673)	0.904
TBT← BIO+CLIN+KL	0.54	0.69	0.47	0.614 (0.566 - 0.661)	0.437
TBT← BIO+CLIN+JSNM	0.55	0.69	0.48	0.622 (0.575 - 0.669)	0.173
TBT← BIO+CLIN+KL+JSNM	0.55	0.69	0.46	0.629 (0.582 - 0.675)	0.025
ΔTBT	0.56	0.69	0.56	0.615 (0.568 - 0.662)	0.680
TBT+ΔTBT	0.58	0.71	0.52	0.672 (0.627 - 0.717)	0.075
TBT+ΔTBT← BIO	0.59	**0.72**	**0.53**	0.676 (0.631 - 0.721)	0.033
TBT+ΔTBT← CLIN	0.59	**0.72**	0.52	0.672 (0.627 - 0.717)	0.062
TBT+ΔTBT← KL	0.58	0.71	0.52	0.669 (0.624 - 0.714)	0.100
TBT+ΔTBT← JSNM	0.58	0.71	0.52	0.675 (0.630 - 0.720)	0.051
TBT+ΔTBT← BIO+CLIN	0.59	**0.72**	0.52	0.674 (0.629 - 0.719)	0.030
TBT+ΔTBT← BIO+KL	0.59	0.71	0.52	0.673 (0.628 - 0.718)	0.046
TBT+ΔTBT← BIO+JSNM	0.59	**0.72**	**0.53**	0.676 (0.631 - 0.721)	0.026
TBT+ΔTBT← BIO+CLIN+ KL	0.59	**0.72**	0.52	0.671 (0.626 - 0.716)	0.023
TBT+ΔTBT← BIO+CLIN+JSNM	0.59	**0.72**	0.52	0.675 (0.630 - 0.720)	0.045
TBT+ΔTBT← BIO+CLIN+KL+JSNM	**0.60**	**0.72**	0.52	0.678 (0.633 - 0.723)	0.011
TBTM← CLIN+KL+JSNM	0.51	0.67	0.40	0.589 (0.542 - 0.637)	0.057
ΔTBTM← CLIN+KL+JSNM	0.51	0.67	0.50	0.567 (0.518 - 0.616)	0.025
TBTM+ΔTBTM← CLIN+KL+JSNM	0.52	0.68	0.44	0.614 (0.567 - 0.661)	0.537
TBTL← CLIN+KL+JSNM	0.51	0.67	0.44	0.566 (0.518 - 0.615)	0.019
ΔTBTL← CLIN+KL+JSNM	0.51	0.67	0.45	0.590 (0.542 - 0.639)	0.160
TBTL+ΔTBTL← CLIN+KL+JSNM	0.53	0.68	0.52	0.596 (0.547 - 0.645)	0.249
TBTC← CLIN+KL+JSNM	0.51	0.67	0.44	0.570 (0.522 - 0.619)	0.022
ΔTBTC← CLIN+KL+JSNM	0.51	0.67	0.50	0.572 (0.522 - 0.622)	0.045
TBTC+ΔTBTC← CLIN+KL+JSNM	0.52	0.67	0.52	0.580 (0.531 - 0.629)	0.062

* refers to the reference model. The model with the descriptor on the left of (←) is adjusted for the descriptor(s) on the right of (←). The highest BACC, PPV, NPV and AUC values obtained are in bold.

Table S3: Secondary analysis: Prediction of all progression using TBT descriptors adjusted for a set of clinical, radiographic and/or biochemical parameters.

Model	BACC	PPV	NPV	AUC (95%CI)	p-value
TBT← CLIN+KL+JSNM*	0.59	0.59	0.58	0.628 (0.574 - 0.682)	-
ΔTBT← CLIN+KL+JSNM	0.58	0.58	0.57	0.628 (0.574 - 0.682)	0.996
TBT+ΔTBT← CLIN+KL+JSNM	0.63	0.64	0.63	0.684 (0.632 - 0.736)	0.022
TBT	0.58	0.59	0.57	0.621 (0.566 - 0.675)	0.668
TBT← BIO	0.59	0.60	0.58	0.632 (0.578 - 0.686)	0.690
TBT← BIO+CLIN	0.59	0.60	0.58	0.629 (0.575 - 0.684)	0.818
TBT← BIO+KL	0.59	0.59	0.58	0.629 (0.575 - 0.683)	0.877
TBT← BIO+JSNM	0.59	0.59	0.58	0.629 (0.575 - 0.683)	0.891
TBT← BIO+CLIN+KL	0.59	0.59	0.58	0.628 (0.574 - 0.683)	0.755
TBT← BIO+CLIN+JSNM	0.59	0.59	0.58	0.624 (0.570 - 0.678)	0.901
TBT← BIO+CLIN+KL+JSNM	0.58	0.59	0.58	0.627 (0.574 - 0.681)	0.614
ΔTBT	0.59	0.59	0.58	0.628 (0.574 - 0.682)	0.988
TBT+ΔTBT	**0.64**	0.64	0.63	0.685 (0.633 - 0.736)	0.047
TBT+ΔTBT← BIO	**0.64**	0.64	0.63	**0.691** (0.639 - 0.742)	0.018
TBT+ΔTBT← CLIN	0.63	0.63	0.62	0.678 (0.626 - 0.730)	0.066
TBT+ΔTBT← KL	0.63	0.64	0.63	0.680 (0.628 - 0.732)	0.068
TBT+ΔTBT← JSNM	0.63	0.64	0.63	0.682 (0.631 - 0.734)	0.050
TBT+ΔTBT← BIO+CLIN	0.63	0.64	0.63	0.683 (0.631 - 0.734)	0.037
TBT+ΔTBT← BIO+KL	0.63	0.64	0.63	0.688 (0.637 - 0.740)	0.025
TBT+ΔTBT← BIO+JSNM	**0.64**	**0.65**	**0.64**	0.688 (0.637 - 0.740)	0.022
TBT+ΔTBT← BIO+CLIN+ KL	0.63	0.63	0.62	0.680 (0.628 - 0.732)	0.042
TBT+ΔTBT← BIO+CLIN+JSNM	0.63	0.64	0.63	0.681 (0.629 - 0.733)	0.046
TBT+ΔTBT← BIO+CLIN+KL+JSNM	0.63	0.64	0.62	0.684 (0.632 - 0.735)	0.023
TBTM← CLIN+KL+JSNM	0.53	0.53	0.52	0.553 (0.497 - 0.609)	0.007
ΔTBTM← CLIN+KL+JSNM	0.53	0.53	0.52	0.538 (0.482 - 0.594)	0.005
TBTM+ΔTBTM← CLIN+KL+JSNM	0.53	0.53	0.52	0.570 (0.514 - 0.625)	0.062
TBTL← CLIN+KL+JSNM	0.55	0.55	0.55	0.566 (0.510 - 0.622)	0.035
ΔTBTL← CLIN+KL+JSNM	0.55	0.55	0.54	0.570 (0.514 - 0.626)	0.060
TBTL+ΔTBTL← CLIN+KL+JSNM	0.55	0.55	0.55	0.588 (0.533 - 0.644)	0.191
TBTC← CLIN+KL+JSNM	0.53	0.54	0.53	0.559 (0.504 - 0.615)	0.028
ΔTBTC← CLIN+KL+JSNM	0.55	0.56	0.55	0.574 (0.518 - 0.630)	0.102
TBTC+ΔTBTC← CLIN+KL+JSNM	0.55	0.55	0.54	0.572 (0.517 - 0.628)	0.083

* refers to the reference model. The model with the descriptor on the left of (←) is adjusted for the descriptor(s) on the right of (←). The highest BACC, PPV, NPV and AUC values obtained are in bold.

Table S4: Secondary analysis: Prediction of radiographic progression using TBT descriptors adjusted for a set of clinical, radiographic and/or biochemical parameters.

Model	BACC	PPV	NPV	AUC (95%CI)	p-value
TBT← CLIN+KL+JSNM*	0.57	0.45	0.78	0.709 (0.653 - 0.765)	-
ΔTBT← CLIN+KL+JSNM	0.60	0.50	0.79	0.742 (0.688 - 0.795)	0.332
TBT+ΔTBT← CLIN+KL+JSNM	0.65	0.51	**0.82**	0.779 (0.731 - 0.827)	0.012
TBT	0.56	0.50	0.77	0.652 (0.591 - 0.713)	0.005
TBT← BIO	0.55	0.44	0.77	0.650 (0.590 - 0.711)	0.003
TBT← BIO+CLIN	0.57	0.46	0.78	0.699 (0.641 - 0.757)	0.319
TBT← BIO+KL	0.55	0.45	0.77	0.644 (0.583 - 0.705)	0.001
TBT← BIO+JSNM	0.56	0.46	0.77	0.664 (0.605 - 0.723)	0.009
TBT← BIO+CLIN+KL	0.57	0.46	0.78	0.694 (0.635 - 0.752)	0.352
TBT← BIO+CLIN+JSNM	0.58	0.47	0.78	0.702 (0.645 - 0.759)	0.136
TBT← BIO+CLIN+KL+JSNM	0.57	0.45	0.78	0.708 (0.652 - 0.764)	0.198
ΔTBT	0.56	0.52	0.77	0.671 (0.612 - 0.731)	0.374
TBT+ΔTBT	0.63	0.49	0.81	0.752 (0.700 - 0.804)	0.191
TBT+ΔTBT← BIO	0.63	0.49	0.81	0.759 (0.707 - 0.810)	0.131
TBT+ΔTBT← CLIN	0.65	0.51	0.82	0.773 (0.724 - 0.822)	0.025
TBT+ΔTBT← KL	0.63	0.48	0.81	0.749 (0.697 - 0.801)	0.230
TBT+ΔTBT← JSNM	0.64	0.49	0.81	0.753 (0.702 - 0.805)	0.172
TBT+ΔTBT← BIO+CLIN	**0.66**	**0.52**	**0.82**	**0.783** (0.736 - 0.831)	0.009
TBT+ΔTBT← BIO+KL	0.63	0.48	0.81	0.754 (0.703 - 0.806)	0.171
TBT+ΔTBT← BIO+JSNM	0.63	0.49	0.81	0.760 (0.709 - 0.811)	0.116
TBT+ΔTBT← BIO+CLIN+ KL	0.65	0.51	**0.82**	0.779 (0.731 - 0.828)	0.012
TBT+ΔTBT← BIO+CLIN+JSNM	0.65	0.51	**0.82**	0.781 (0.733 - 0.829)	0.014
TBT+ΔTBT← BIO+CLIN+KL+JSNM	0.65	0.51	**0.82**	0.779 (0.731 - 0.828)	0.011
TBTM← CLIN+KL+JSNM	0.52	0.39	0.76	0.654 (0.594 - 0.713)	0.038
ΔTBTM← CLIN+KL+JSNM	0.53	0.44	0.76	0.678 (0.616 - 0.739)	0.368
TBTM+ΔTBTM← CLIN+KL+JSNM	0.56	0.52	0.77	0.683 (0.623 - 0.742)	0.423
TBTL← CLIN+KL+JSNM	0.52	0.43	0.76	0.653 (0.589 - 0.716)	0.022
ΔTBTL← CLIN+KL+JSNM	0.53	0.42	0.76	0.678 (0.618 - 0.738)	0.325
TBTL+ΔTBTL← CLIN+KL+JSNM	0.55	0.50	0.77	0.676 (0.615 - 0.736)	0.256
TBTC← CLIN+KL+JSNM	0.51	0.35	0.75	0.652 (0.590 - 0.713)	0.032
ΔTBTC← CLIN+KL+JSNM	0.52	0.46	0.75	0.647 (0.588 - 0.706)	0.041
TBTC+ΔTBTC← CLIN+KL+JSNM	0.52	0.38	0.76	0.653 (0.595 - 0.712)	0.034

* refers to the reference model. The model with the descriptor on the left of (←) is adjusted for the descriptor(s) on the right of (←). The highest BACC, PPV, NPV and AUC values obtained are in bold.

Table S5: Secondary analysis: Prediction of symptomatic progression using TBT descriptors adjusted for a set of clinical, radiographic and/or biochemical parameters.

Model	BACC	PPV	NPV	AUC (95%CI)	p-value
TBT← CLIN+KL+JSNM*	0.53	0.40	0.76	0.643 (0.583 - 0.703)	-
ΔTBT← CLIN+KL+JSNM	0.53	0.39	0.76	0.661 (0.602 - 0.720)	0.615
TBT+ΔTBT← CLIN+KL+JSNM	0.60	0.49	**0.79**	0.710 (0.654 - 0.766)	0.027
TBT	0.51	0.31	0.75	0.620 (0.560 - 0.679)	0.276
TBT← BIO	0.52	0.35	0.75	0.629 (0.568 - 0.690)	0.454
TBT← BIO+CLIN	0.53	0.42	0.76	0.651 (0.591 - 0.711)	0.110
TBT← BIO+KL	0.52	0.35	0.75	0.636 (0.576 - 0.696)	0.670
TBT← BIO+JSNM	0.52	0.36	0.75	0.631 (0.571 - 0.692)	0.487
TBT← BIO+CLIN+KL	0.53	0.41	0.76	0.649 (0.589 - 0.708)	0.475
TBT← BIO+CLIN+JSNM	0.53	0.42	0.76	0.646 (0.587 - 0.706)	0.030
TBT← BIO+CLIN+KL+JSNM	0.53	0.40	0.76	0.645 (0.586 - 0.705)	0.027
ΔTBT	0.51	0.33	0.75	0.626 (0.567 - 0.686)	0.701
TBT+ΔTBT	0.59	0.50	0.78	0.698 (0.641 - 0.756)	0.132
TBT+ΔTBT← BIO	0.60	0.52	**0.79**	0.699 (0.641 - 0.758)	0.106
TBT+ΔTBT← CLIN	0.59	0.49	**0.79**	0.711 (0.655 - 0.766)	0.044
TBT+ΔTBT← KL	0.59	0.49	0.78	0.696 (0.638 - 0.754)	0.144
TBT+ΔTBT← JSNM	0.59	0.49	0.78	0.697 (0.639 - 0.754)	0.142
TBT+ΔTBT← BIO+CLIN	**0.61**	**0.51**	**0.79**	**0.718** (0.662 - 0.774)	0.013
TBT+ΔTBT← BIO+KL	0.60	**0.51**	**0.79**	0.700 (0.642 - 0.758)	0.094
TBT+ΔTBT← BIO+JSNM	0.59	0.50	**0.79**	0.700 (0.642 - 0.758)	0.095
TBT+ΔTBT← BIO+CLIN+ KL	0.60	0.50	**0.79**	0.713 (0.657 - 0.769)	0.016
TBT+ΔTBT← BIO+CLIN+JSNM	0.60	0.50	**0.79**	0.715 (0.660 - 0.771)	0.019
TBT+ΔTBT← BIO+CLIN+KL+JSNM	0.60	0.49	**0.79**	0.710 (0.654 - 0.766)	0.027
TBTM← CLIN+KL+JSNM	0.50	0.07	0.75	0.566 (0.503 - 0.630)	0.018
ΔTBTM← CLIN+KL+JSNM	0.50	0.13	0.74	0.601 (0.538 - 0.664)	0.230
TBTM+ΔTBTM← CLIN+KL+JSNM	0.50	0.31	0.75	0.618 (0.557 - 0.679)	0.463
TBTL← CLIN+KL+JSNM	0.51	0.37	0.75	0.594 (0.533 - 0.655)	0.088
ΔTBTL← CLIN+KL+JSNM	0.50	0.01	0.74	0.584 (0.519 - 0.649)	0.096
TBTL+ΔTBTL← CLIN+KL+JSNM	0.50	0.27	0.75	0.618 (0.558 - 0.678)	0.427
TBTC← CLIN+KL+JSNM	0.51	0.54	0.75	0.570 (0.505 - 0.634)	0.006
ΔTBTC← CLIN+KL+JSNM	0.50	0.22	0.75	0.571 (0.509 - 0.633)	0.041
TBTC+ΔTBTC← CLIN+KL+JSNM	0.52	0.49	0.75	0.588 (0.526 - 0.650)	0.055

* refers to the reference model. The model with the descriptor on the left of (←) is adjusted for the descriptor(s) on the right of (←). The highest BACC, PPV, NPV and AUC values obtained are in bold.
